# Treatment Compliance Among People With Type 2 Diabetes in Rural Tamil Nadu, India: Prevalence, Associated Factors, and a Comparison of Public and Private Care

**DOI:** 10.7759/cureus.113729

**Published:** 2026-07-31

**Authors:** Elanchezhian D, Joy Bazroy, Ravindhara Bharathi G, Sengadhirvendhan R, Albertine R

**Affiliations:** 1 Preventive and Social Medicine, Pondicherry Institute of Medical Sciences, Pondicherry, IND; 2 Internal Medicine, Pondicherry Institute of Medical Sciences, Pondicherry, IND

**Keywords:** glycaemic control, india, medication adherence, rural health, treatment compliance, type 2 diabetes

## Abstract

Background: Treatment compliance determines whether therapy for type 2 diabetes succeeds in practice, yet it is poorly characterized in rural India, where free public services and fee-charging private services operate side by side. We estimated the prevalence of treatment compliance, identified its independent correlates, and compared compliance between government and private facilities in a rural community of Tamil Nadu.

Methods: In this community-based cross-sectional study, 220 adults with type 2 diabetes on treatment for at least three months were selected by simple random sampling. Compliance was measured with the nine-item Hill-Bone Medication Adherence Scale and dichotomized at the sample median. Independent correlates were identified by binary logistic regression, and the most recent fasting and post-prandial blood glucose values were retrieved as an objective correlate. Associations were tested with the chi-squared and Fisher exact tests and the Mann-Whitney U test, with p < 0.05 considered significant.

Results: Only 34.1% of participants were compliant. Compliant patients had markedly lower fasting glucose (143 vs. 181 mg/dL) and post-prandial glucose (236 vs. 295 mg/dL; both p < 0.001). In multivariable analysis, only a diabetes duration exceeding 10 years independently predicted compliance (adjusted odds ratio 3.39, 95% confidence interval (CI) 1.55-7.40; p = 0.002); the univariate associations with age and travel time did not persist after adjustment. Compliance was almost identical in government and private facilities (34.4% vs. 33.7%; p = 0.907), even though 24.5% of private-care users reported being unable to afford treatment, and it was unrelated to sex, education, occupation, or socioeconomic class.

Conclusions: Two-thirds of rural adults with type 2 diabetes were non-compliant, and non-compliance was mirrored in significantly worse glycemic control. Public and private care achieved equal compliance despite an unequal cost burden, and only longer disease experience distinguished compliant patients. Duration-sensitive adherence support that engages the recently diagnosed, delivered across both care streams, is needed.

## Introduction

Type 2 diabetes has become one of the defining chronic disease challenges of the century. An estimated 537 million adults were living with diabetes worldwide in 2021, a figure projected to reach 783 million by 2045, with more than three-quarters of the burden now concentrated in low- and middle-income countries [1]. India carries the second-largest burden after China, with roughly 74 million affected adults, and the recent Indian Council of Medical Research-India Diabetes (ICMR-INDIAB) survey placed the national prevalence at 11.4% [1,2]. In such settings, the clinical benefit of proven pharmacological and lifestyle therapies depends less on the availability of medicines than on whether patients sustain them over years of asymptomatic disease.

Poor treatment compliance is the point at which effective therapy fails in practice. Non-compliance accelerates disease progression, provokes avoidable hospital admissions, and inflates the cost of care, consequences that fall hardest on populations with limited resources [3,4]. The World Health Organization frames compliance as a multidimensional behavior shaped by socioeconomic, health-system, condition-related, therapy-related, and patient-related influences, rather than a matter of individual willpower alone [5]. Indian community studies have repeatedly found that close to half of people with diabetes fail to adhere to prescribed treatment, implicating financial constraints, weak family support, low literacy, forgetfulness, and the type of health facility attended, although estimates vary widely by region and by how adherence is measured [6-9].

Rural populations warrant particular attention. They combine rising diabetes prevalence with fragmented health-seeking behavior, seasonal and occupational demands that interrupt daily dosing, and a mixed economy of free government services alongside fee-charging private practitioners. How compliance compares between these two streams of care, and whether the affordability advantage of public provision translates into better adherence, has seldom been quantified in a single rural community. Most available estimates also rely on medication counts alone.

We therefore conducted a community-based cross-sectional study in a rural block of Chengalpattu District, Tamil Nadu, to estimate the prevalence of treatment compliance among adults with type 2 diabetes, to identify the sociodemographic, clinical, and health-system factors associated with it, and to compare compliance between patients attending government and private facilities.

## Materials and methods

Study design and setting

This community-based cross-sectional study was conducted in the field-practice area of the Rural Health Training Center at Chunampet, Chengalpattu District, Tamil Nadu, which is served by the Department of Community Medicine of a tertiary teaching institution. Reporting follows the Strengthening the Reporting of Observational Studies in Epidemiology (STROBE) guidance for cross-sectional studies [10]. The study formed the quantitative component of a larger mixed-methods project; the qualitative findings are reported separately.

Participants

Eligible participants were community-dwelling adults older than 30 years with physician-diagnosed type 2 diabetes who had been on pharmacotherapy for at least three months. Individuals who were critically ill or who remained unavailable after two consecutive household visits were excluded. A sampling frame of patients with type 2 diabetes was drawn from the center's family folders, and 220 participants were selected by simple random sampling using the lottery method.

Sample size

The sample size was calculated using the standard formula for estimating a single proportion, n = Z²P(1 − P)/d², where Z = 1.96 (corresponding to 95% confidence), P is the anticipated proportion of treatment compliance, and d is the absolute precision. Assuming a compliance proportion of 52% from an earlier South Indian study [11] and 7% absolute precision, this gave n = (1.96 x 1.96) x 0.52 x 0.48/(0.07 x 0.07) = 195.8, approximately 196. Allowing for a 10% non-response rate gave a final target of 220 participants.

Variables and measurements

Trained interviewers administered a structured questionnaire at the participant's home in Tamil. Data comprised sociodemographic characteristics (age, sex, education, occupation, and per-capita income), diabetes history (duration of diabetes), health-seeking behavior (place, frequency, and convenience of consultation, blood-glucose monitoring, and travel time to and difficulty in reaching the facility), out-of-pocket costs, and lifestyle. Socioeconomic status was classified using the updated modified B.G. Prasad scale [12]. Because government facilities dispense medicines free of charge, consultation and medicine costs and affordability were recorded only for participants attending private facilities (n = 98).

Treatment compliance

Compliance was measured with the nine-item Hill-Bone Medication Adherence Scale [13], each item scored on a four-point scale. All nine items describe barrier behaviors (for example, forgetting or deciding not to take medicine), so that a higher total denotes better adherence. Total scores were dichotomized at the sample median (median score 34): scores above the median were classified as compliant and scores at or below the median as non-compliant.

Objective glycemic correlate

To examine whether self-reported compliance corresponded to a clinical outcome, the most recent fasting and post-prandial capillary blood-glucose values recorded in participants' treatment documents were retrieved (available for 201 and 205 participants, respectively). These were routine clinic measurements rather than standardized study assays and are reported as a supportive correlate.

Statistical analysis

Data were entered in EpiData v3.1 (EpiData Association, Odense, Denmark) and analyzed in IBM SPSS Statistics v21 (IBM Corp., Armonk, NY, USA). Categorical variables are summarized as frequencies and percentages, and continuous variables as mean ± standard deviation or median (interquartile range). Associations between compliance and categorical exposures were tested with the Pearson chi-squared test, the chi-squared test for trend for ordered exposures, or the Fisher exact test where expected counts were small; blood-glucose values were compared between compliance groups using the Mann-Whitney U test. To identify independent correlates of compliance, variables associated at p < 0.20 on univariate testing, together with the treating facility (a pre-specified variable of interest), were entered into a multivariable binary logistic-regression model, from which adjusted odds ratios (aORs) with 95% confidence intervals (CIs) were derived. Model fit was assessed with the Hosmer-Lemeshow test. A two-sided p-value below 0.05 was considered statistically significant.

Ethical approval

The protocol was approved by the Institutional Ethics Committee of Pondicherry Institute of Medical Sciences (IEC No. RC/2022/59). Written informed consent was obtained from every participant after provision of a participant information sheet in English and Tamil, in accordance with the ICMR National Ethical Guidelines. Participation was voluntary and uncompensated, and no invasive procedures were involved.

## Results

Participant characteristics

All 220 selected participants were interviewed. Their mean age was 54 ± 11.5 years, and the majority were women (59.5%). More than a third had received no formal schooling (37.3%), and most were engaged in unskilled work (46.4%) or were unemployed (34.5%). Under the modified B.G. Prasad classification, 56.4% belonged to the highest socioeconomic class. The full sociodemographic, clinical, and health-seeking profile is shown in Table 1. More than half of participants attended government facilities (55.5%) and the remainder private facilities (44.5%). Among the 98 private-facility users, monthly medicine spending (recorded for 94) was most often ₹501-₹1,000 (46.8%), and 24.5% reported being unable to afford consultation or medicines.

**Table 1:** Sociodemographic, clinical, treatment, and health-seeking characteristics of participants (N = 220). Data are n (%) unless otherwise stated. Affordability was assessed only among the 98 private-facility users; monthly medicine spending was recorded for 94 of them (most commonly ₹501-₹1,000, 46.8%). SD: standard deviation

Characteristic	n (%)
Age, years (mean ± SD)	54 ± 11.5
Sex	
Female	131 (59.5)
Male	89 (40.5)
Education	
No formal schooling	82 (37.3)
Primary	38 (17.3)
Middle	57 (25.9)
High school	35 (15.9)
Graduate	8 (3.6)
Occupation	
Unemployed	76 (34.5)
Unskilled	102 (46.4)
Semiskilled	36 (16.4)
Skilled	6 (2.7)
Socioeconomic class (modified B.G. Prasad scale)	
Class I	124 (56.4)
Class II	48 (21.7)
Class III	29 (13.2)
Class IV	12 (5.5)
Class V	7 (3.2)
Duration of diabetes	
≤1 year	35 (15.9)
1-3 years	62 (28.2)
3-5 years	48 (21.8)
5-10 years	36 (16.4)
>10 years	39 (17.7)
Treating facility	
Government	122 (55.5)
Private	98 (44.5)
Travel time to facility	
<1/2 hour	118 (53.6)
1/2-1 hour	92 (41.8)
>1 hour	10 (4.5)
Health-seeking behavior and access	
Difficulty in reaching facility (yes)	50 (22.7)
Medication side-effects (yes)	18 (8.2)
Monthly physician consultation (yes)	129 (58.6)
Lifestyle advice received (yes)	201 (91.4)
Follow-up convenient (yes)	183 (83.2)
Unable to afford care, private users (yes)	24 (24.5)

Prevalence of treatment compliance

Only 75 of 220 participants (34.1%) were classified as compliant on the Hill-Bone scale, leaving 145 (65.9%) non-compliant. A single independent item pointed the same way: 60.3% reported having stopped or missed medication in the preceding two weeks, and this was strongly concordant with the scale; only 18.2% of those who reported a recent lapse were classified as compliant.

Compliance and glycemic control

**Figure 1:**
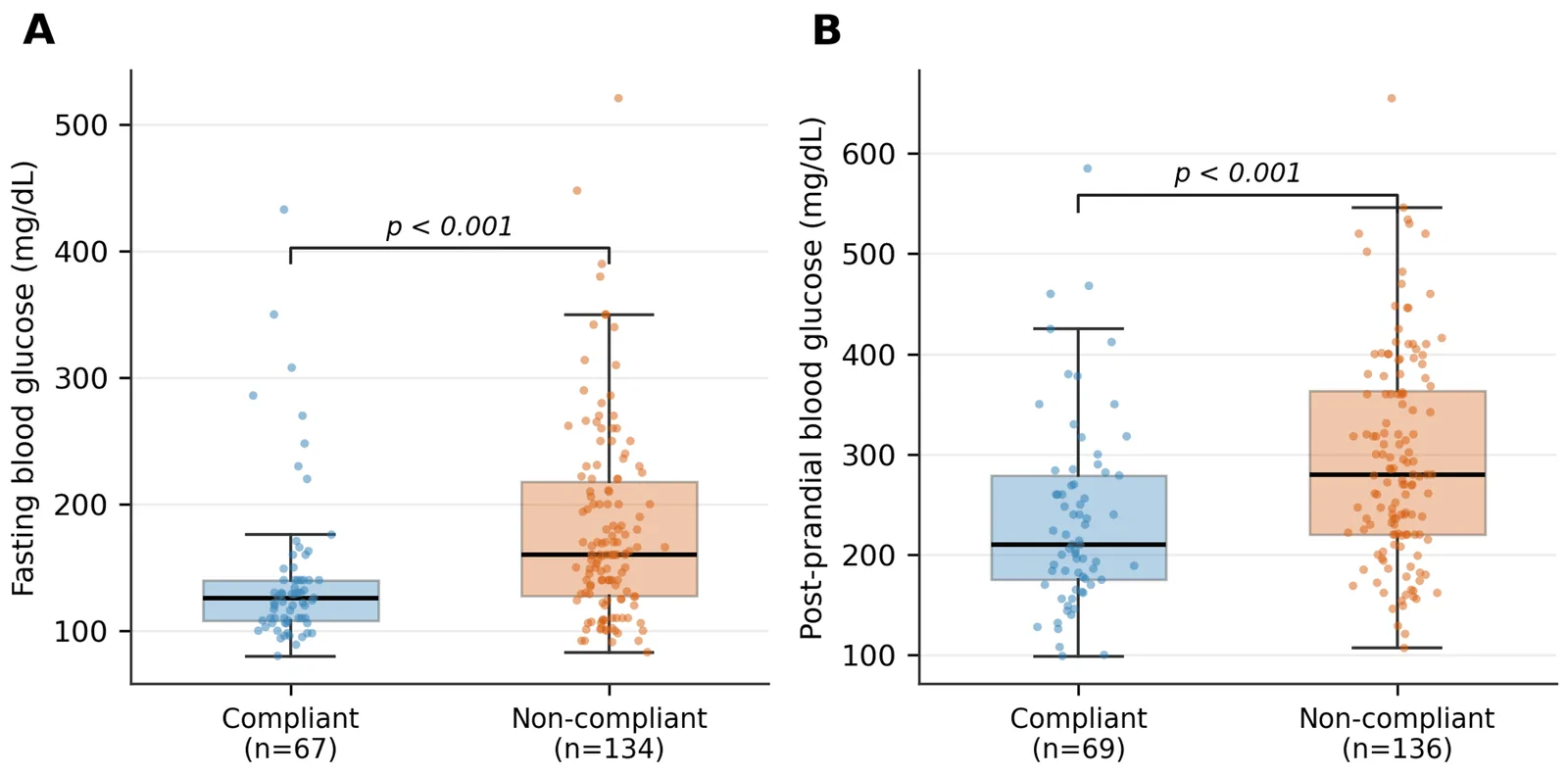
(A, B) Fasting and post-prandial capillary blood glucose by treatment compliance status. Compliant participants had significantly lower glucose on both measures (Mann-Whitney U test, p < 0.001). Values are the most recent routine clinic measurements (fasting n = 201; post-prandial n = 205).

Compliance was mirrored in objective glycemia. Compliant participants had substantially lower fasting glucose (mean 143 ± 64 vs. 181 ± 76 mg/dL; median 126 vs. 160 mg/dL) and post-prandial glucose (236 ± 94 vs. 295 ± 104 mg/dL; median 210 vs. 280 mg/dL) than non-compliant participants (both p < 0.001). The difference in fasting glucose is shown in Figure 1A and that in post-prandial glucose in Figure 1B. The gradient supports the validity of the Hill-Bone classification in this population.

Factors associated with compliance

**Figure 2:**
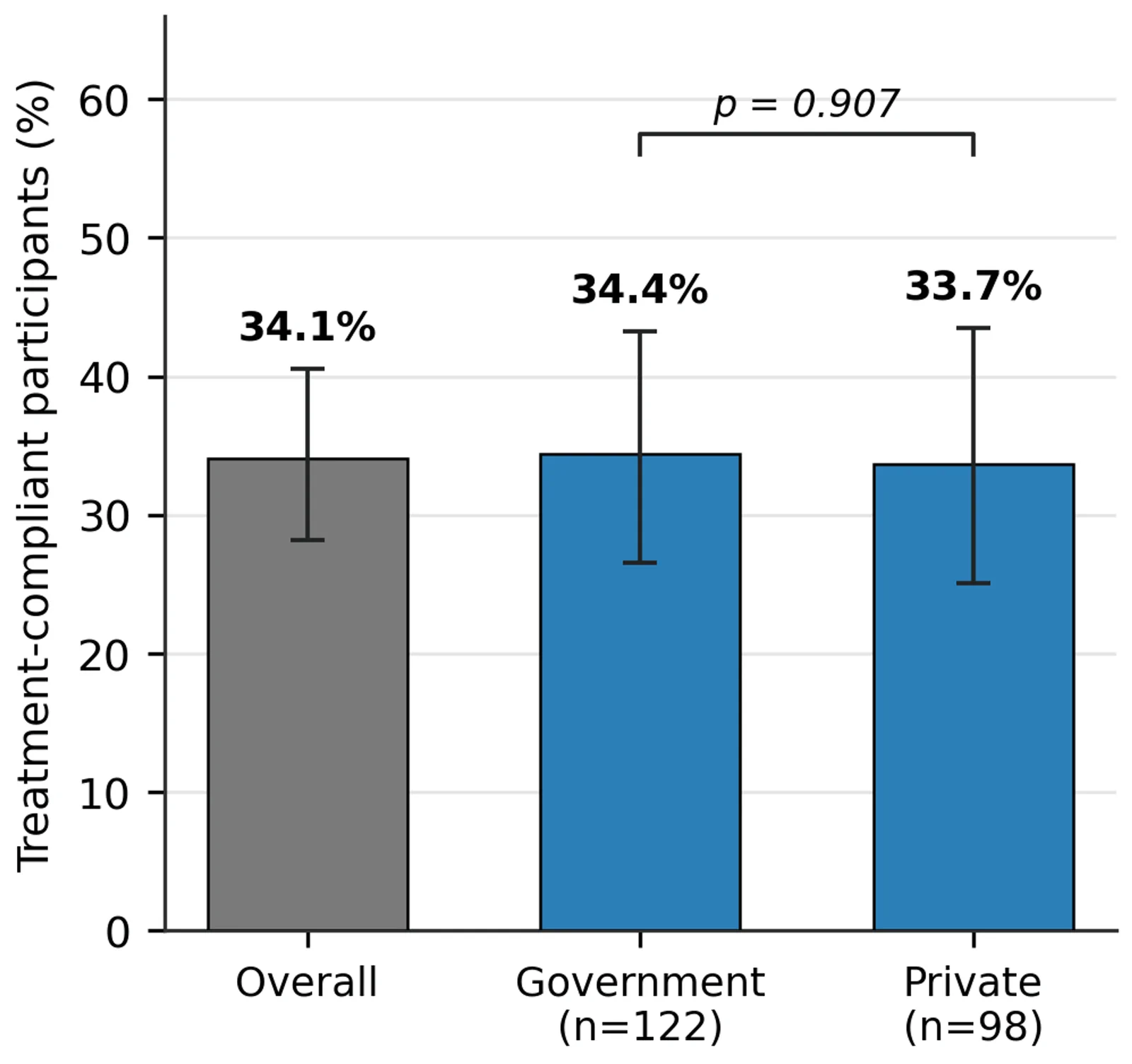
Prevalence of treatment compliance overall and by treating facility. The difference between government and private facilities was not statistically significant (chi-squared test, p = 0.907); error bars are 95% confidence intervals.

On univariate analysis, compliance rose across age groups, from 21.4% in those aged 30-39 years to 56.2% in those aged 70 years and older (test for trend, p = 0.039). Longer disease experience showed the strongest gradient: 61.5% of participants with diabetes for more than 10 years were compliant, against 20.8%-37.1% in shorter-duration groups (χ² = 18.32, p = 0.001). Compliance also differed by travel time, with the small group travelling more than an hour reporting the highest compliance (70.0%; p = 0.032). Compliance was unrelated to sex (p = 0.498), education (p = 0.477), occupation (p = 0.777), socioeconomic class (p = 0.257), difficulty in access (p = 0.507), or, among private-care users, monthly medicine spending (p = 0.260), and was almost identical between government and private facilities (34.4% vs. 33.7%; p = 0.907; Figure 2). These associations are summarized in Table 2.

**Table 2:** Factors associated with treatment compliance on univariate analysis (N = 220). Values are the number (%) of compliant participants within each stratum; stratum denominators are given in Table 1. The p-value shown with each factor is for the comparison across all of its categories. Overall, 75 of 220 participants (34.1%) were compliant. Adjusted odds ratios are given in Table 3. ^†^Chi-squared test for trend. *Diabetes duration χ² = 18.32, df = 4; travel time χ² = 6.90, df = 2; socioeconomic class χ² = 5.31, df = 4.

Factor	Compliant, n (%)
Age group, years (p = 0.039)^†^	
30-39	6 (21.4)
40-49	24 (35.8)
50-59	20 (28.2)
60-69	16 (42.1)
≥70	9 (56.2)
Sex (p = 0.498)	
Female	47 (35.9)
Male	28 (31.5)
Education (p = 0.477)	
No formal schooling	29 (35.4)
Primary	11 (28.9)
Middle	19 (33.3)
High school	11 (31.4)
Graduate	5 (62.5)
Occupation (p = 0.777)	
Unemployed	28 (36.8)
Unskilled	34 (33.3)
Semiskilled	12 (33.3)
Skilled	1 (16.7)
Socioeconomic class (p = 0.257)	
Class I	39 (31.5)
Class II	16 (33.3)
Class III	9 (31.0)
Class IV	7 (58.3)
Class V	4 (57.1)
Duration of diabetes (p = 0.001)*	
≤1 year	13 (37.1)
1-3 years	18 (29.0)
3-5 years	10 (20.8)
5-10 years	10 (27.8)
>10 years	24 (61.5)
Treating facility (p = 0.907)	
Government	42 (34.4)
Private	33 (33.7)
Travel time to facility (p = 0.032)	
<1/2 hour	35 (29.7)
1/2-1 hour	33 (35.9)
>1 hour	7 (70.0)
Difficulty in reaching facility (p = 0.507)	
Yes	19 (38.0)
No	56 (32.9)

**Table 3:** Unadjusted and adjusted odds ratios (ORs) for treatment compliance (binary logistic regression, N = 220). *p-value for the adjusted OR. Model likelihood-ratio p = 0.002; Hosmer-Lemeshow p = 0.16; 75 compliant events. Duration of treatment was excluded owing to collinearity with diabetes duration (Spearman ρ = 0.94). CI: confidence interval

Variable	Unadjusted OR (95% CI)	Adjusted OR (95% CI)	p*
Age (per 10 years)	1.29 (1.01-1.64)	1.14 (0.87-1.49)	0.34
Female (vs. male)	1.22 (0.69-2.16)	1.18 (0.64-2.16)	0.59
Diabetes > 10 years (vs. ≤10)	4.08 (1.98-8.39)	3.39 (1.55-7.40)	0.002
Private facility (vs. government)	0.97 (0.55-1.70)	0.89 (0.48-1.67)	0.72
Travel >1 hour (vs. ≤1 hour)	4.87 (1.22-19.43)	3.01 (0.67-13.48)	0.15

Independent correlates

In the multivariable model (Table 3), only a diabetes duration exceeding 10 years remained independently associated with compliance (aOR 3.39, 95% CI 1.55-7.40; p = 0.002). The univariate associations with age (aOR 1.14 per decade; p = 0.34) and with travel time (aOR 3.01; p = 0.15) were attenuated to non-significance after adjustment, indicating that they were largely explained by disease duration. The treating facility remained unrelated to compliance after adjustment (private vs. government aOR 0.89, 95% CI 0.48-1.67; p = 0.72). The model was statistically significant (likelihood-ratio p = 0.002) with satisfactory calibration (Hosmer-Lemeshow p = 0.16).

## Discussion

In this rural South Indian community, only one in three adults with type 2 diabetes met the threshold for treatment compliance, and non-compliance was therefore the norm rather than the exception. The finding sits within a wide but consistently discouraging range reported across India: compliance near 17% in one clinic-based series [14] and around a third in a tertiary-care cohort from eastern India [15], against roughly a half in several community studies [6,7] and as high as 90% in one rural sample [16], while poor adherence reached 74% in rural Kerala [8]. This spread reinforces that sustaining diabetes therapy, not initiating it, is the binding constraint on control in these settings, and that differences in how adherence is defined and measured account for much of the between-study variation, an argument for standardized instruments such as the Hill-Bone scale used here [13].

The most policy-relevant result is the absence of any difference in compliance between government and private care. Patients attending free public facilities were no more likely to comply than those paying out of pocket privately, even though a quarter of private-care users reported difficulty affording their treatment. This contrasts with reports linking the type of facility to adherence in rural Tamil Nadu [6] and invites two readings. On one hand, publicly funded provision, including home delivery of medicines under state outreach schemes, appears to have neutralized the affordability disadvantage that might otherwise depress adherence among poorer patients, an encouraging signal for universal-access policy. On the other hand, the persistence of two-thirds non-compliance across both streams shows that removing the price barrier is necessary but far from sufficient, and that behavioral, informational, and organizational barriers dominate once cost is addressed.

Compliance was patterned by disease experience rather than by social advantage. After multivariable adjustment, only a diabetes duration beyond 10 years predicted compliance, roughly trebling the odds, while age, sex, education, occupation, socioeconomic class, and the source of care showed no independent association. A comparable influence of disease duration on adherence has been reported from Vellore and from Puducherry [11,17], and the pattern is biologically coherent: accumulating symptoms, complications, and clinical encounters over years may progressively convince patients of the need to persist. The univariate effects of age and travel time disappeared once disease duration was accounted for, a reminder that unadjusted associations in adherence research are readily confounded. The corollary for practice is uncomfortable but clear: the recently diagnosed, who are asymptomatic and not yet convinced, adhere least, precisely when early control would yield the greatest long-term benefit.

A particular strength of these data is that the compliance classification tracked an objective outcome: compliant patients had fasting and post-prandial glucose roughly 40 and 60 mg/dL lower, respectively, than their non-compliant counterparts. Much of the Indian adherence literature rests on self-report alone, and the concordance here between a validated self-report scale, a single independent recall item, and recorded glycemia lends unusual confidence that the two-thirds non-compliance figure reflects a real and clinically consequential gap rather than a measurement artefact; better adherence has likewise been associated with better glycemic control in South Indian and other cross-sectional studies [18,19]. It also reframes non-compliance as a proximate, modifiable cause of the poor control evident in this community, where mean fasting and post-prandial values sat well above target. Despite near-universal receipt of lifestyle advice, the lifestyle dimension of self-management deserves as much attention as medication-taking [20].

Strengths and limitations

The principal strengths of this study are its community basis and probability sampling, which reduce the selection bias inherent in the hospital-based samples that dominate the Indian literature, its use of a validated adherence instrument corroborated by recorded glycemia, and multivariable adjustment to isolate independent correlates. Conducting interviews at home and in the local language improved participation and candor. Several limitations temper interpretation. The cross-sectional design precludes causal inference, and reverse causation cannot be excluded for time-dependent factors such as disease duration. Compliance was self-reported and, despite the convergent evidence, may still be overestimated through social-desirability bias. The blood-glucose values were routine clinic measurements of differing recency rather than standardized study assays, and glycated hemoglobin was available for too few participants to use; the glycemic comparison should therefore be read as supportive rather than definitive. Finally, the study was conducted in a single rural block served by one training center, which may limit generalizability, and the modest number of compliant events constrained the number of variables the regression could support.

## Conclusions

Two-thirds of adults with type 2 diabetes in this rural community were non-compliant with treatment, and that non-compliance was mirrored in significantly worse glycemic control. Compliance was no better under free public care than under paid private care, even though affordability strained a quarter of private-care users, and the only independent marker of compliance was longer disease duration rather than any socioeconomic advantage, leaving the recently diagnosed as the most vulnerable group. Publicly funded access appears to have equalized the cost playing field but has not, on its own, produced adequate adherence. Community-based, duration-sensitive adherence support that engages patients early after diagnosis and operates across both public and private care is needed to close this gap.
